# Wet Nurses and Lactation

**Published:** 1848-08

**Authors:** 

**Affiliations:** Fulda


*Wet Nurses and Lactation.* By Dr. Schneider of Fulda.—Dr. Schneider inveighs strongly against corsets and other pectoral portions of female dress, as a great means of preventing females from making good nurses. The pressure on the breasts of young women weakens these organs, depresses and hardens the nipples, and obstructs the lactiferous ducts. It is, according to Dr. S., a very rare thing to find a perfect nurse. If we cannot be sure of finding an unexceptionable nurse, Dr. S. recommends Zwierlein's plan of taking a goat as a substitute. He has himself made upwards of thirty trials with children, whose mothers were unfit to suckle their offspring, and has in all of them obtained successful results by making the children draw the milk directly from the teats of the goat. No animal is so well fitted for this purpose as the goat. We are to select a young tame healthy animal, take care not to feed it too well, especially if the child is strong and plethoric, have it carefully rubbed down and cleaned, and give it frequently salt with its meat. A small wooden platform to which one or two steps lead, is to be erected close to the wall of the room; it must not be polished, that the animal's feet may not slide. To the wall, or to a beam of wood, a leather strap is fastened, which when the goat has mounted on the stage, can be easily secured round its neck, and to prevent its disturbing the child by moving backwards or forwards, its hind legs are to be secured in like manner. Lastly, at the end of the stage there is to be placed a tin dish with a cover, to catch any discharge from the animal.—*Prager Vierel-jahrschrift.*

